# Connectome harmonic decomposition tracks the presence of disconnected consciousness during ketamine-induced unresponsiveness

**DOI:** 10.1016/j.bja.2024.12.036

**Published:** 2025-02-10

**Authors:** Milan Van Maldegem, Jakub Vohryzek, Selen Atasoy, Naji Alnagger, Paolo Cardone, Vincent Bonhomme, Audrey Vanhaudenhuyse, Athena Demertzi, Oceane Jaquet, Mohamed Ali Bahri, Pablo Nunez, Morten L. Kringelbach, Emmanuel A. Stamatakis, Andrea I. Luppi

**Affiliations:** 1 Department of Clinical Neurosciences, University of Cambridge, Cambridge, UK; 2 Department of Physiology, Development & Neuroscience, University of Cambridge, Cambridge, UK; 3 Division of Anaesthesia, University of Cambridge, Cambridge, UK; 4 Centre for Eudaimonia and Human Flourishing, Department of Psychiatry, University of Oxford, Oxford, UK; 5 Centre for Brain and Cognition, Computational Neuroscience Group, Department of Information and Communication Technologies, Universitat Pompeu Fabra, Barcelona, Spain; 6 Centre for Music in the Brain, Aarhus University, Aarhus, Denmark; 7 Coma Science Group, GIGA-Consciousness, University of Liege, Liege, Belgium; 8 Centre du Cerveau, University Hospital of Liege, Liege, Belgium; 9 Anaesthesia and Perioperative Neuroscience, GIGA-Consciousness, University of Liege, Liege, Belgium; 10 Department of Anesthesia and Intensive Care Medicine, University Hospital of Liege, Liege, Belgium; 11 Conscious Care Lab, GIGA-Consciousness, University of Liege, Liege, Belgium; 12 Algology Interdisciplinary Centre, University Hospital of Liege, Liege, Belgium; 13 Physiology of Cognition Lab, GIGA-CRC Human Imaging Unit, University of Liege, Liege, Belgium; 14 Psychology and Neuroscience of Cognition Research Unit, University of Liege, Liege, Belgium; 15 GIGA-CRC Human Imaging Unit, University of Liege, Liege, Belgium; 16 Division of Information Engineering, University of Cambridge, Cambridge, UK; 17 St John's College, University of Cambridge, Cambridge, UK

**Keywords:** connectome harmonics, consciousness, dissociative anaesthesia, functional MRI, ketamine dreams, unresponsiveness

## Abstract

**Background:**

Ketamine, in doses suitable to induce anaesthesia in humans, gives rise to a unique state of unresponsiveness accompanied by vivid experiences and sensations, making it possible to disentangle the correlated but distinct concepts of conscious awareness and behavioural responsiveness. This distinction is often overlooked in the study of consciousness.

**Methods:**

The mathematical framework of connectome harmonic decomposition (CHD) was used to view functional magnetic resonance imaging (fMRI) signals during ketamine-induced unresponsiveness as distributed patterns across spatial scales. The connectome harmonic signature of this particular state was mapped onto signatures of other states of consciousness for comparison.

**Results:**

An increased prevalence of fine-grained connectome harmonics was found in fMRI signals obtained during ketamine-induced unresponsiveness, indicating higher granularity. After statistical assessment, the ketamine sedation harmonic signature showed alignment with signatures of LSD-induced (fixed effect =0.0113 [0.0099, 0.0127], *P*<0.001) or ketamine-induced (fixed effect =0.0087 [0.0071, 0.0103], *P*<0.001) psychedelic states, and misalignment with signatures seen in unconscious individuals owing to propofol sedation (fixed effect =–0.0213 [–0.0245, –0.0181], *P*<0.001) or brain injury (fixed effect =–0.0205 [–0.0234, –0.0178], *P*<0.001).

**Conclusions:**

The CHD framework, which only requires resting-state fMRI data and can be applied retrospectively, has the ability to track alterations in conscious awareness in the absence of behavioural responsiveness on a group level. This is possible because of ketamine's unique property of decoupling these two facets, and is important for consciousness and anaesthesia research.

**Editor's key points**

• Ketamine produces a unique state of unresponsiveness characterised by vivid experiences and sensations, in contrast to other common general anaesthetics.
• This phenomenon was used to disentangle the imaging signatures of conscious awareness and behavioural responsiveness by functional MRI (fMRI) and high-resolution EEG.
• Connectome harmonic decomposition was used to analyse fMRI signals during ketamine-induced unresponsiveness across spatial scales and mapped onto signatures of other states of consciousness.
• The ketamine signature mapped well onto the signatures for psychedelic drugs such as LSD and psilocybin, and might be related to the psychedelic-like reports of volunteers during ketamine sedation.
• Connectome harmonic decomposition analysis could be used to track consciousness in subjects who appear unconscious based on behavioural responsiveness.

In 1966, the first clinical study investigating ketamine (called Cl-581) as a human anaesthetic showed that it could produce strong analgesia along with a unique state of altered consciousness, and that its limited duration of effect could be safely extended with repeated administration.^1^ Later studies found that increasing doses (from 0.3 to 1 mg kg^−1^ h^−1^) initially result in antidepression, followed by pain relief and altered perception, such as sensations of detachment, visual distortions, and confused thinking.^2^, ^3^, ^4^, ^5^, ^6^ Loss of behavioural responsiveness is usually observed only with administration of high doses (1–6 mg kg^−1^ h^−1^).^4^^,^^7^^,^^8^

At the molecular level, ketamine inhibits hyperpolarisation-activated cyclic nucleotide-gated K^+^ channel 1 (HCN1),^9^^,^^10^ and acts as a noncompetitive antagonist of the *N*-methyl-*D*-aspartate (NMDA) receptor by blocking and reducing channel open frequency.^11^ This results mainly in suppression of GABAergic inhibitory cortical interneurones, generating an overall increase in brain excitability.^12^, ^13^, ^14^ Ketamine also influences other types of interneurones, causing spontaneously active cortical neurones to become silent and previously silent neurones to become active.^15^ The roles of NMDA receptors^11^^,^^16^, ^17^, ^18^ and other targets^8^^,^^19^ in ketamine's multiple actions are under active investigation.

At the systems neuroscience level, the mechanisms of ketamine sedation show major differences from those of other general anaesthetics such as propofol, xenon, and sevoflurane. For example, ketamine preserves metabolism in the thalamus,^20^ suppresses sleep-promoting regions of the hypothalamus,^21^ activates arousal-promoting areas of the brainstem and diencephalon,^21^ maintains cortical functional complexity,^22^, ^23^, ^24^, ^25^ and increases EEG and magnetoencephalographic (MEG) gamma activity in the cortex.^26^, ^27^, ^28^, ^29^, ^30^, ^31^, ^32^ Conversely, GABAergic anaesthetics generally induce opposite changes.^33^, ^34^, ^35^ Ketamine is also thought to interrupt information transfer between cortical regions while maintaining the function of sensory networks and subcortical areas.^36^, ^37^, ^38^, ^39^ Despite some similarities with more traditional anaesthetics,^38^^,^^40^^,^^41^ ketamine induces a specific large-scale organisation of functional activity throughout the brain, which might be associated with its distinct molecular targets.^42^

Another unique characteristic of ketamine-induced unresponsiveness is the high prevalence of disconnected consciousness (e.g. dreams, when there are subjective experiences, but with little relationship to the external environment) compared with states induced by more traditional anaesthetics.^25^^,^^43^, ^44^, ^45^, ^46^ This is a convenient property, allowing researchers to disentangle the correlated but distinct concepts of behavioural responsiveness and conscious awareness.^47^^,^^48^ Until recently, the ability to provide coherent responses to external stimuli served as the primary criterion for assessing consciousness in noncommunicating patients and nonhuman animals.^49^, ^50^, ^51^ Yet, this assumes that unresponsive states uniformly signify unconsciousness, despite evidence indicating that vivid (but environmentally disconnected) experiences can manifest within such states. For example, dreams and other sensations have been observed during various stages of sleep,^52^, ^53^, ^54^ and even under general anaesthesia.^25^^,^^45^^,^^46^^,^^54^, ^55^, ^56^ Moreover, after severe brain injury, some patients remain unresponsive at the bedside yet show some level of disconnected consciousness through neuroimaging.^57^, ^58^, ^59^ It thus seems crucial to distinguish the brain dynamics associated with conscious awareness from those related to behavioural responsiveness.

Multiple lines of evidence have shown that human consciousness relies on a dynamic repertoire of brain activity,^60^, ^61^, ^62^, ^63^, ^64^, ^65^, ^66^, ^67^, ^68^, ^69^ which prompts inquiry into how a static network of anatomical connections, the human connectome, can give rise to these complex dynamics.^70^, ^71^, ^72^, ^73^, ^74^, ^75^ Early exploration has developed our understanding of the loss of consciousness and its signatures: as consciousness fades, the patterns of correlation among regional BOLD time series (functional connectivity [FC]) tend to mirror more closely the pattern of anatomical connections between these regions, resulting in a more global functional organisation of brain activity.^60^^,^^61^^,^^76^, ^77^, ^78^, ^79^ In contrast, brain activity patterns tend to be more fine-grained and localised in altered states of consciousness induced by psychedelic drugs or meditation.^80^, ^81^, ^82^, ^83^, ^84^, ^85^ One way to investigate these functional organisation patterns is through the mathematical framework of connectome harmonic decomposition (CHD)^86^; an overview of this framework is in Fig. 1. Briefly, CHD is analogous to the well-known Fourier transform, but in space rather than in time. The Fourier transform quantifies the prevalence of different temporal frequencies in a time series. Analogously, connectome harmonics reflect the prevalence of different spatial granularities in a pattern of brain activity, from coarse-grained (global) to more fine-grained (localised).

**Fig 1:**
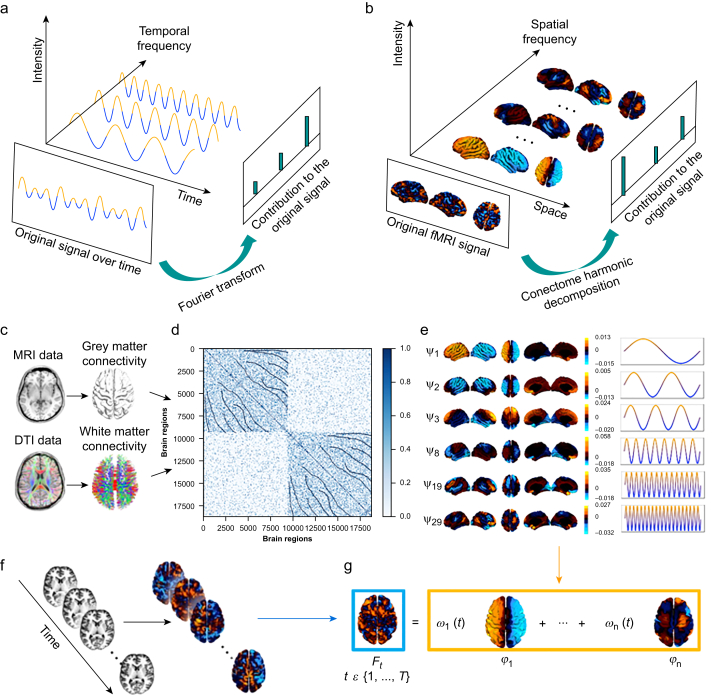
Overview of connectome harmonic decomposition (CHD) of resting-state functional MRI (fMRI) data. (a) The widely used Fourier analysis decomposes a time series signal into sinusoidal basis wave functions (‘temporal harmonics’) of different frequency. Temporal harmonics with high frequencies represent rapidly changing signals, where data points close in time can have different values. Low-frequency temporal harmonics correspond to signals that change slowly over time, resulting in temporally adjacent data points having similar values, indicating a stronger time-dependent nature of the signal. (b) CHD decomposes a signal originating in the spatial domain (expressed as BOLD activation across distinct spatial points throughout the cortex) into ‘harmonic modes’ of the human structural connectome, termed ‘connectome harmonics’, each having a distinct spatial granularity (from coarse-grained to fine-grained). Like temporal harmonics reflect the prevalence of different temporal frequencies in the signal, so connectome harmonics reflect the prevalence of different spatial granularities in a pattern of brain activity, from global (coarse-grained) to local (fine-grained). (c) A high-resolution human connectome is obtained from structural and diffusion MRI. (d) Local and long-distance connections are integrated to construct the connectivity matrix. (e) The graph Laplacian of this high-resolution connectome is subsequently broken down into its eigenvectors *ϕ*_1…*n*_ (harmonic modes) and their corresponding eigenvalues *λ*_1…*n*_ (spatial frequencies with ascending granularity) by solving the following equation: $\Delta_{G}\phi_{k}\left(v_{i}\right)=\lambda_{k}\phi_{k}\left(v_{i}\right)$. (f) At each timepoint *t*ϵ {*1, …, T*}, fMRI data is projected from Montreal Neurological Institute volumetric space to FreeSurfer surface space. (g) CHD summarising fMRI time series as a linear summation of individual harmonics (*ϕ*_*k*_) and their weights, *ω*_*k*_(*t*), at each timepoint *t*ϵ {*1, …, T*}. DTI, diffusion tensor imaging.

Luppi and colleagues^83^ have suggested that the harmonic signature obtained by CHD reliably tracks alterations of consciousness, with an increased contribution of coarse-grained (global) harmonics in propofol sedation and disorders of consciousness (DOC) and an increased contribution of fine-grained (localised) patterns in typical (LSD and psilocybin) and atypical (low doses of ketamine) psychedelic states. To make sure that they were not tracking mere behavioural responsiveness, these researchers used a dataset of patients (*N*=22) who met the diagnostic criteria for DOC, of which some (*N*=8) demonstrated conscious awareness by successfully completing mental imagery tasks while in an MRI scanner.^87^ The results showed an increased contribution of coarse-grained patterns in patients who did not show signs of consciousness compared with those who did. However, it is difficult to know with absolute certainty that the unresponsive patients had no form of consciousness, as some of them might have been conscious but unable to do the task for a variety of reasons (e.g. impairments in language processing, hearing difficulties).

Ketamine deep sedation has the unique property of *temporarily* suppressing behavioural responsiveness in individuals while preserving some degree of conscious awareness,^25^^,^^43^, ^44^, ^45^, ^46^ enabling them to report experiences, if any, during the sedated period. The dataset used in this study includes such (anecdotal) reports, collected via phone calls after study completion, indicating that all eight volunteers experienced some form of conscious awareness despite being behaviourally unresponsive (Supplementary Table S1).^88^ Although these reports were not gathered using a structured questionnaire, similar dream-like experiences during ketamine-induced unresponsiveness have been documented in another study that used standardised surveys,^25^ lending further support to the observations in the present dataset.

By applying the framework of CHD to this rare dataset, we aimed to build on the results of Luppi and colleagues,^83^ and further disentangle the concepts of behavioural responsiveness and conscious awareness in the neuroscientific study of consciousness. The original investigation using the dataset presented here provided evidence of specific disruptions in FC between key resting-state networks (e.g. default-mode network [DMN], salience network) during ketamine sedation. We sought to build on these results, taking a whole-brain perspective, rather than network-based perspective, on how ketamine-induced unresponsiveness alters brain activity in healthy volunteers. Specifically, we sought to test two opposite hypotheses. First, the connectome harmonic signature obtained by CHD tracks behavioural responsiveness, in which case brain activity during ketamine-induced unresponsiveness should show a more coarse-grained (global) pattern of brain activity compared with wakefulness. Second, the connectome harmonic signature tracks consciousness, in which case we should see equally or more fine-grained (localised) activity patterns in ketamine-induced unresponsiveness compared with wakefulness. We also compared the CHD signatures with those produced by LSD, subanaesthetic ketamine, propofol, and DOC.^83^

## Methods

### Subjects

Data were collected at the University Hospital of Liege (Liege, Belgium, registered at EudraCT 2010-023016-13) as described in the original publication.^88^ In short, 14 right-handed volunteers (age 19–31 yr, five women) were recruited through an online advertisement (internet forum) and underwent a medical interview and physical examination before participation. Of these, 13 volunteers completed the imaging session; five had to be excluded from further analyses owing to excessive agitation and movements, leaving eight for analysis.

### Protocol

A detailed description of the study protocol has been published.^88^ Briefly, volunteers were instructed to refrain from consuming solids for a minimum of 6 h and liquids for 2 h before the experimental session. Upon entering the investigation unit, a thorough review of potential contraindications to participation was conducted, including criteria for both anaesthesia and MRI, using a comprehensive checklist. After structural MRI acquisition, subjects were removed from the scanner, and 64 EEG scalp electrodes were placed to allow for simultaneous EEG recording during functional MRI (fMRI) data acquisition (Brain Amp® magnetic resonance compatible acquisition setup; Brain Products GmbH, Gilching, Germany). Our main focus was on fMRI data analysis owing to the high spatial resolution needed to answer our research question, but we also included results pertaining to the EEG.

Ketamine was delivered by i.v. through a computer-controlled infusion device, which consisted of a laptop computer connected to an infusion pump. The pharmacokinetic model used to regulate the pump was the Domino model,^89^ which has been validated and shown to exhibit satisfactory predictive performance.^90^ For each alteration in ketamine concentration, a 5-min equilibration period was allowed upon reaching the target.^88^ The Ramsay Scale (RS)^91^ and the University of Michigan Sedation Scale (UMSS)^92^ were used to assess depth of sedation. Evaluation occurred directly before and after each fMRI data acquisition sequence. Volunteers were instructed to firmly squeeze the hand of the investigator, with the command reiterated twice. To ensure close monitoring of volunteers, an investigator remained inside the MRI scan room throughout the process.

Initial fMRI data acquisition was performed in the absence of ketamine. Ketamine infusion was then started, with target concentration increased in steps of 0.5 μg ml^−1^ until a level of sedation corresponding to RS 3–4 or UMSS 1–2 was reached (light sedation, S1). After the 5-min equilibration period, a second sequence of data acquisition occurred, consisting of the same sequence of events as during wakefulness. Ketamine target concentration was then further increased in steps of 0.5 μg ml^−1^ until RS 5–6 or UMSS 4 (ketamine-induced unresponsiveness, S2), and the same sequence of data acquisition was again performed. Table 1 shows ketamine concentrations and other parameters.

**Table 1:** Relevant parameters for baseline (Awake), light sedation (Ketamine S1), and deep sedation (Ketamine S2). In addition, results of the one-way anova for repeated measures are provided. ∗Significantly lower than S1 and S2 as assessed by Tukey honestly significantly difference tests on three means with 14 degrees of freedom. †Significantly lower than S2 as assessed by the same *post hoc* comparison tests. Adapted, with permission, from Bonhomme and colleagues.^88^ N/A, nonapplicable; NS, nonsignificant.

Parameter	Awake	Ketamine S1	Ketamine S2	Statistics
Mean blood pressure (mm Hg), mean (sd)	94 (11)∗	107 (13)†	120 (16)	F_(2, 14)_ =19.14, *P*<0.0001
Heart rate (beats min^−1^), mean (sd)	70 (14)∗	92 (18)	108 (10)	F_(2, 14)_ =27.97, *P*<0.0001
Peripheral saturation in oxygen (%), mean (sd)	99 (0)	99 (0)	98 (1)	F_(2, 14)_ =2.70, NS
Estimated ketamine plasma concentration (μg ml^−1^), mean (range)	N/A	0.75 (0.5–1.5)	2 (1.5–2.5)	N/A
Measured arterial carbon dioxide partial pressure (kPa), mean (sd)	5.47 (0.53)	5.2 (0.93)	4.93 (1.07)	F_(2, 14)_ =1.22, NS
Ramsay sedation score, median (range)	2 (2–2)	2 (2–3)	6 (4–6)	N/A
University of Michigan Sedation Score, median (range)	0 (0–0)	1.5 (0–2)	4 (3–4)	N/A

Because ketamine has a long elimination half-life, and to limit time spent in the fMRI scanner for the volunteer, the temporal order of those clinical states was not randomised. For the same reason, a recovery condition could not be achieved. After these acquisitions, infusion of ketamine was stopped, and the subject was removed from the fMRI scanner for recovery. The presence of dreaming during ketamine infusion was checked through a phone call at distance from the experimental session.^88^

### MRI data acquisition

The acquisition procedures are described in detail in the original study.^88^ Briefly, the experiment was performed on a 3T Siemens Allegra scanner (Siemens AG, Munich, Germany; Echo Planar Imaging sequence using 32 slices; repetition time (TR) =2460 ms; echo time =40 ms; field of view =220 mm; voxel size =3.45×3.45×3 mm; matrix size =64×64×32). For each volunteer and each condition, 300 functional volumes were acquired. In addition, a high-resolution structural T1 image was obtained at the beginning of the experiment for co-registration to the functional data.

### Functional MRI preprocessing and denoising

Preprocessing and denoising followed the same methods as in previous publications, including work that used the CHD analysis, to ensure consistency.^65^^,^^66^^,^^83^ For clarity and constancy of reporting, the same wording is used as in those publications where applicable. The functional imaging data were preprocessed using a standard pipeline implemented within the SPM12-based (http://www.fil.ion.ucl.ac.uk/spm) toolbox CONN (http://www.nitrc.org/projects/conn), version 17f.^93^ The pipeline consisted of the following steps: removal of the first five scans to allow magnetisation to reach steady-state; functional realignment and motion correction; slice-timing correction to account for differences in time of acquisition between slices; identification of outlier scans for subsequent regression by means of the quality assurance/artifact rejection software *art* (http://nitrc.org/projects/artifact_detect); structure-function co-registration using each participant's high-resolution T1-weighted image; spatial normalisation to Montreal Neurological Institute (MNI-152) standard space with 2 mm isotropic resampling resolution, using the segmented grey matter image, together with an *a priori* grey matter template.

To reduce noise as a result of cardiac and motion artifacts, the anatomical CompCor method of denoising was used,^94^ also implemented within the CONN toolbox.^93^ Linear detrending was also applied, and the subject-specific denoised BOLD signal time series were band-pass filtered to eliminate both low-frequency drift effects and high-frequency noise, thus retaining temporal frequencies between 0.008 and 0.09 Hz. This band-pass filtering pertains to temporal frequencies, which are distinct from the spatial frequencies obtained from CHD (described below).^83^

### Connectome reconstruction

The following workflow was as described.^80^ A high-resolution human structural connectome was obtained from diffusion tensor imaging and structural MRI data from an independent sample of 10 Human Connectome Project (HCP) subjects (six females, age 23–35 yr). For each subject, the cortical surfaces of each hemisphere at the interface of white and grey matter were reconstructed using FreeSurfer (http://freesurfer.net), which resulted in a representation of 18 715 cortical surface vertices per participant. When each subject's diffusion imaging and cortical surface data were co-registered, every vertex of the reconstructed cortical surface served as a centre to initialise eight seeds for deterministic tractography, performed using the MrDiffusion tool (http://white.stanford.edu/newlm/index.php/MrDiffusion). Tracking was terminated when fractional anisotropy (FA) was below a threshold of 0.3, with a minimum tract length of 20 mm, and a maximum angle of 30° between consecutive tracking steps.

The structural connectivity (SC) of each participant was represented as a binary adjacency matrix, denoted as A, with each cortical surface vertex considered a node. For every pair of nodes *i* and *j* out of the *n*=18 715 total nodes, *A*_*ij*_ was set to 1 if a white matter tract connected them, as estimated from the deterministic tractography step described above, or if they were adjacent in the grey matter cortical surface representation. If neither long-range nor short-range connections between *i* and *j* existed, *A*_*ij*_ was set to 0. The whole process resulted in a symmetric (undirected) binary matrix as previously described.^80^ Subsequently, the individual adjacency matrices of the 10 HCP participants were averaged to derive a group-average matrix $\bar{A}$, which represents a typical structural connectome. Finally, the degree matrix *D* of the graph was defined, where each element $D_{ij}$ represents the sum of connections for node *i* across all nodes *j*: $D_{ii}=\sum_{j=1}^{n}\bar{A_{ij}}$.

### Connectome harmonics extraction

Following the procedure of Atasoy and colleagues,^80^ the graph Laplacian $\Delta_{G}$ was computed on the group-average adjacency matrix $\bar{A}$, described above, in order to estimate the discrete counterpart of the Laplace operator Δ.^95^

ΔG=D−12LD−12,withL=D−A¯

Subsequently, the connectome harmonics $\phi_{k},k\in \left\{1,\ldots ,18,715\right\}$ were calculated by solving the following eigenvalue problem:

$$\Delta_{G}\phi_{k}\left(v_{i}\right)=\lambda_{k}\phi_{k}\left(v_{i}\right),\forall v_{i}\in V,with\;0<\;\lambda_{1}<\;\lambda_{2}<\ldots <\;\lambda_{n}$$

where $\lambda_{k}$, k ∈ {1, …, *n*} is the corresponding eigenvalue of the eigenfunction $\phi_{k}$, *V* is the set of cortical surface vertices, and *n* represents the number of vertices. The frequencies associated with each connectome harmonic are in the spatial rather than the temporal domain, and should not be confused with the temporal frequencies identified by Fourier transform in the temporal domain (e.g. time series denoising).

### Connectome harmonic decomposition of functional MRI data

At each timepoint *t* ∈ {1, …, *T*}, which corresponds to one TR, the preprocessed and denoised fMRI data were projected onto cortical surface coordinates by means of the HCP Workbench *volume-to-surface-mapping* tool.^80^ Subsequently, the spatial pattern of cortical activity over vertices *v* at time *t*, denoted as *F*_*t*_*(v)*, was decomposed as a linear combination of the set of connectome harmonics $\Psi ={\left\{\phi_{k}\right\}}_{k=1}^{N}$:

$$F_{t}=\omega_{1}\left(t\right)\phi_{1}+\omega_{2}\left(t\right)\phi_{2}+\ldots +\omega_{n}\left(t\right)\phi_{n}=\sum_{k=1}^{n}\omega_{k}\left(t\right)\phi_{k}\left(v\right)$$

with the contribution $\omega_{k}\left(t\right)$ of each connectome harmonic $\phi_{k}$ at time *t* being estimated as the projection (dot product) of the fMRI data *F*_*t*_*(v)* unto $\phi_{k}:{\;\omega }_{k}\left(t\right)=\left\langle F_{t},\phi_{k}\right\rangle$.

The transition from low-frequency (coarse-grained) to high-frequency (fine-grained) connectome harmonics indicates a move from coarse-grained to more fine-grained activity patterns.^80^^,^^86^ Other authors have interpreted the same shift as a departure of functional activity from the SC beneath it.^96^

### Connectome harmonic power and energy

Following the procedure of Atasoy and colleagues,^80^ the magnitude of contribution of each harmonic $\phi_{k}$, k ∈ {1, …, *n*} at any given timepoint *t*, called its ‘power’, is computed as the amplitude of its contribution: *P(*$\phi_{k}$, t*)=|* $\omega_{k}\left(t\right)$ *|*.

In turn, the normalised frequency-specific contribution of each harmonic $\phi_{k}$, k ∈ {1, …, *n*} at timepoint *t*, called ‘energy’, is estimated by combining the strength of activation (*|* $\omega_{k}\left(t\right)$ *|*^*2*^) of a particular connectome harmonic with its own intrinsic energy (${\lambda_{k}}^{2})$.

$$E\left(\phi_{k},t\right)={\left|\omega_{k}\left(t\right)\right|}^{2}\lambda_{k}^{2}$$

Consequently, total brain energy at time *t* is given by

$$E_{total}\left(t\right)=\sum_{k=1}^{n}{\left|\omega_{k}\left(t\right)\right|}^{2}{\lambda_{k}}^{2}=|\left|{\Delta F}_{t}\left(v\right)\right||²$$

Finally, a binned energy spectrum across subjects and timepoints is construed by discretising the energy of connectome harmonics into 15 logarithmically spaced frequency-specific bins, following previous work showing that this procedure can successfully highlight the connectome harmonic signatures of altered states of consciousness.^80^^,^^81^^,^^83^^,^^85^

### Data-driven extraction of multivariate connectome harmonic signatures

Partial Least Squares (PLS), also recognised as Projection on Latent Structures, is a multivariate statistical method used for modelling the relationship between one or multiple target variables (Y) and a set of predictor variables (X). The main goal is to find a set of latent variables, also known as principal components, that capture the maximal covariance with each other. X was the matrix of 15 binned energy values for each subject (averaged over timepoints), and Y was the vector of binary classification between the two states (awake *vs* S1 and awake *vs* S2). Because of the binary nature of Y, this analysis can be seen as an application of Partial Least Squares Discriminant Analysis (PLS-DA).^97^ The first principal component represents the most discriminative pattern present in the data in terms of distinguishing between two states, which we term the state's multivariate signature (MVS) as described.^83^

### Diversity of connectome harmonic repertoire

A diverse repertoire of connectome harmonics can be defined as a repertoire in which different harmonic modes contribute in different degrees to brain activity (Supplementary Fig. S3a). This means that there is neither one dominating mode, corresponding to a periodic oscillation, nor equal contribution of every mode to the signal, corresponding to white noise. To quantify the connectome harmonic diversity, the entropy of the distribution of connectome harmonic power (absolute strength of contribution to the cortical activation pattern) across all 18 715 harmonics was calculated (for each timepoint of each subject). To deal with continuous data, the Kozachenko approximation was used, as implemented in the Java Information Dynamics Toolbox (JIDT; http://jlizier.github.io/jidt/).^98^ With continuous variables, entropy can adopt negative values^99^; however, interpretations remain the same: a more entropic distribution corresponds to a more diverse connectome harmonic repertoire.

### EEG preprocessing

A total of 64 EEG channels were recorded. Channels M1, M2, nose1, and node2 were removed from the analysis, resulting in 60 channels (locations of the remaining electrodes shown in Supplementary Fig. S1). Gradient artifacts caused by the fMRI signal were removed using an average artifact template subtraction (AAS) algorithm as implemented in the FMRIB plugin for the EEGLAB toolbox (https://fsl.fmrib.ox.ac.uk/eeglab/fmribplugin/) provided by the University of Oxford Centre for Functional MRI of the Brain (FMRIB). We applied AAS by averaging the signals aligned with the slice event markers automatically generated by the hardware synchronisation between the EEG amplifier and the MR scanner clock. In some cases, the slice event markers were absent from the recording. We inspected the raw EEG to manually mark the timing of the first slice and then generated the remaining slice markers based on the TR and number of slices. The removal procedure was carried out by a subtraction of the average artifact template from each channel,^100^ followed by an optimal basis set (OBS) of principal components for the removal of artifact residuals.^101^ After the removal of the gradient artifact, data were downsampled to 250 Hz. EEG data were then band-pass filtered between 1 and 70 Hz. The fMRI slice selection frequency (13 Hz for this study) and its harmonics and AC power line noise (50 Hz) were removed by band rejection filtering (1 Hz bandwidth). The ballistocardiogram (BCG) artifact was also removed by AAS and OBS of principal components using the FMRIB plugin. The heartbeat was detected using simultaneously-recorded electrocardiogram (ECG) data. A constant delay of 210 ms between the cardiac event markers and the main BCG peak was assumed for all AAS-based methods, which is the default value in the FMRIB plugin.^102^ The four principal components that explained most of the artifact waveform variance were automatically selected and regressed-out from the data. Independent component analysis was then performed using the EEGLAB *runica* algorithm. Independent components were manually inspected for each subject to remove residual gradient artifacts, BCG artifacts, and eye movements.

### Lempel–Ziv complexity

We used Lempel–Ziv complexity (LZC) to analyse the complexity of EEG brain activity, as described.^103^ The algorithm was applied to the envelope of the preprocessed EEG time series. In order to convert the time series to binary sequences, the envelopes were thresholded using the median value. The complexity of the sequence is proportional to the number of binary sequences or ‘words’ found on the sequence. Contrary to the procedure used by Schartner and colleagues,^103^ we applied the LZC algorithm to each EEG channel separately in order to get complexity maps of the whole brain, and LZC values were normalised by the theoretical upper bound of complexity of binary sequences of length N ($N/\log_{2}\left(N\right)$).^104^

### Statistical analysis and reproducibility

To be consistent with previous study,^83^ statistical significance of the difference between conditions (wakefulness *vs* ketamine sedation) was assessed using linear mixed-effects models (MATLAB function: *fitlme*), considering ‘condition’ as a fixed effect and ‘subjects’ as random effects. Timepoints were also included as random effects, nested within subjects, when a single measurement was obtained for each $t_{i}$ (i∈{1,…,300}). Results are reported in terms of the fixed effect, alongside the upper and lower bounds of the 95% confidence interval, and the associated *P*-value. Subject variables (i.e. age and biological sex) were included as covariates of no interest. The false discovery rate for multiple comparisons across 15 frequency bins of harmonic energy was controlled utilising the Benjamini–Hochberg correction,^105^ Spearman's nonparametric rank-based *ρ* was used for correlation, and χ^2^ tests were used to evaluate the statistical dependence between variables (to identify potential gradients). All analyses, except for χ^2^, were two-sided, with an *alpha* threshold of 0.05.

## Results

We utilised the CHD framework to extract the harmonic signatures underlying ketamine-induced unresponsiveness. We primarily report comparisons between the deepest stage of ketamine sedation (S2) and wakefulness (Awake), as these are sufficient to test our hypotheses. Additional comparisons, including those between the lighter sedative state (S1) and wakefulness (Awake), and between the two different steps of ketamine sedation (S1 *vs* S2), are available in the Supplementary materials and briefly discussed.

The same analysis was applied using an alternative reconstruction of the human connectome at higher spatial resolution. This reconstruction was obtained by aggregating data from 985 subjects from the HCP, providing one of the most comprehensive representations of the human structural connectome currently available.^83^ Results with this 985 HCP connectome can also be found in the Supplementary materials.

### Ketamine-induced unresponsiveness increases energy of brain activity

The total energy of each connectome harmonic was computed by merging its activation power (the absolute contribution to the cortical fMRI activity at each timepoint) with its intrinsic energy depending on spatial granularity (eigenvalue). By utilising these metrics, the overall impact of ketamine-induced unresponsiveness (S2) was investigated across the entire range of connectome harmonics. The analysis showed that the total energy of all 18 715 harmonics averaged across all timepoints increased under ketamine-induced unresponsiveness (S2) compared with normal wakefulness (Awake), *t*(14)=2.8208, *P*=0.0257 (repeated-measures *t*-test; Supplementary Fig. S1b). The increase was also seen for a lower dose of ketamine (S1), *t*(14)=3.2204, *P*=0.0146 (repeated-measures *t*-test; Supplementary Fig. S2a); there was no significant difference in ketamine-induced unresponsiveness (S2) compared with lighter sedation (S1), *t*(14)=2.0563, *P*=0.0788 (repeated-measures *t*-test; Supplementary Fig. S2c).

### Ketamine-induced unresponsiveness is characterised by an increased contribution of fine-grained harmonics

**Fig 2:**
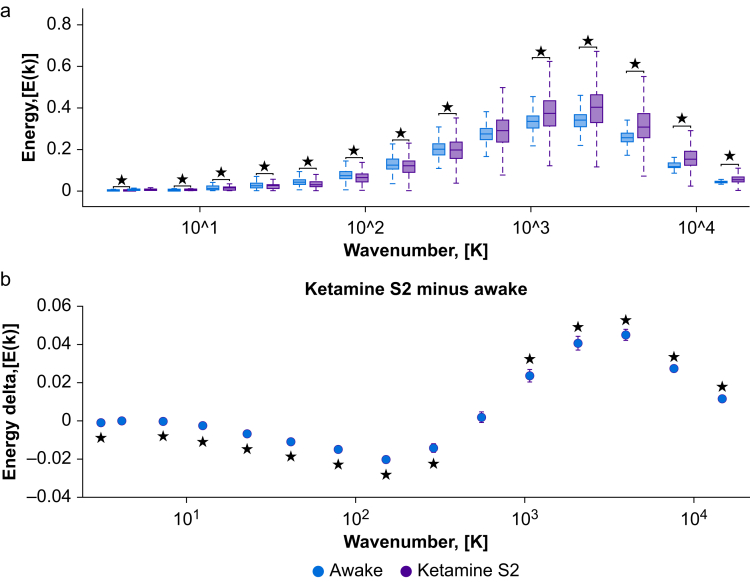
Connectome harmonic signature of ketamine-induced unresponsiveness. (a) The binned energy spectrum across subjects and timepoints (*N*=8, 300 timepoints each) with ketamine-induced unresponsiveness (S2) as target state and wakefulness (Awake) as reference state. (b) Statistical estimates from linear mixed-effect modelling between wakefulness (Awake) and ketamine-induced unresponsiveness (S2), treating condition as a fixed effect and subjects as random effects. Timepoints were also incorporated as random effects, nested within subjects. A brain surface projection illustrating the connectome harmonic pattern associated with each spatial granularity bin is displayed above the respective bin. These projections are averaged over the constituent spatial granularities within each bin. *∗p*<0.05, false discovery rate corrected across 15 frequency bins.

We examined which connectome harmonics, if any, exhibited heightened contributions to brain activity under ketamine-induced unresponsiveness (S2) compared with wakefulness (Awake). To achieve this, we analysed changes in brain dynamics induced by ketamine at different spatial granularities, by initially discretising the connectome harmonic spectrum into 15 levels of wavenumbers in the logarithmic space. Then, the energy changes within each bin of the harmonic spectrum were investigated for each condition and subject separately. An overview of this procedure can be found in Fig. 2 and in the Methods.

When comparing ketamine deep sedation (S2) with normal wakefulness (Awake) using linear mixed-effect modelling, significant changes were observed in the energy levels of nearly all quantised levels of spatial granularity (*P*<0.05 with the Benjamini–Hochberg correction; Fig. 2b). An increase was found in connectome harmonic energy across a broad range of fine-grained harmonics (wave numbers >200 out of 18 715 connectome harmonics), whereas the energy of connectome harmonics decreased for wave numbers <200 (Fig. 2b). This pattern was also found for lighter ketamine sedation (S1) compared with wakefulness (Awake), and even between the two different doses (S2>S1) (Supplementary Fig. S3). Both doses of ketamine showed greater diversity in the range of connectome harmonics compared with normal wakefulness (Supplementary Fig. S4). Taken together, the results indicate that the contribution of fine-grained harmonics to the fMRI BOLD signal seems to gradually increase with higher concentrations of ketamine, at least up to a certain point.

### Relating ketamine harmonic signatures to other altered states of consciousness

It is possible to generalise connectome harmonic patterns across datasets, thereby establishing the harmonic signature of both unconsciousness and the psychedelic state.^83^ To achieve this, PLS-DA was used to comprehensively consider the entire spectrum of connectome harmonic changes simultaneously. This data-driven technique allowed the researchers to extract multivariate patterns of connectome harmonic energy, referred to as MVSs, that effectively differentiate between pairs of conditions. The first principal component derived from PLS-DA represents the most discriminative pattern in the data, distinguishing subjects belonging to different states of consciousness. Luppi and colleagues^83^ showed that this approach revealed two mirror-reversed multivariate patterns characterising the loss of consciousness (propofol anaesthesia and DOC) and the psychedelic state (LSD and subanaesthetic ketamine). The same approach was also used to show that the connectome harmonic signature of the serotonergic psychedelic DMT matches those of LSD and subanaesthetic ketamine.^85^

In the current study, each subject's harmonic signature was projected onto various MVSs obtained by Luppi and colleagues,^83^ after which the value of this projection was compared between conditions (ketamine-induced unresponsiveness [S2] and wakefulness [Awake]). The projection was executed using the dot product, a mathematical operator that measures the extent to which two vectors point in the same direction, giving a positive value for vectors that align in directionality and a negative value for vectors that point in the opposite direction. Here, a positive result indicates an alignment between two different MVSs, whereas a negative result indicates misalignment. An outcome of zero means that both MVSs (vectors) are orthogonal and not related. Important to note is that the sign is of primary importance here, and not the magnitude of the measure: we want to know whether two patterns are aligned (positive sign, indicating similar effect on the brain) or misaligned (negative sign, indicating opposite effects on the brain). The statistical tests provide us with a confidence measure that shows how certain we can be of our sign; the lower the *P*-value, the less probable it is that the real (population-based) dot product between the vectors of the two conditions is zero, and thus has a positive or negative sign.

**Fig 3:**
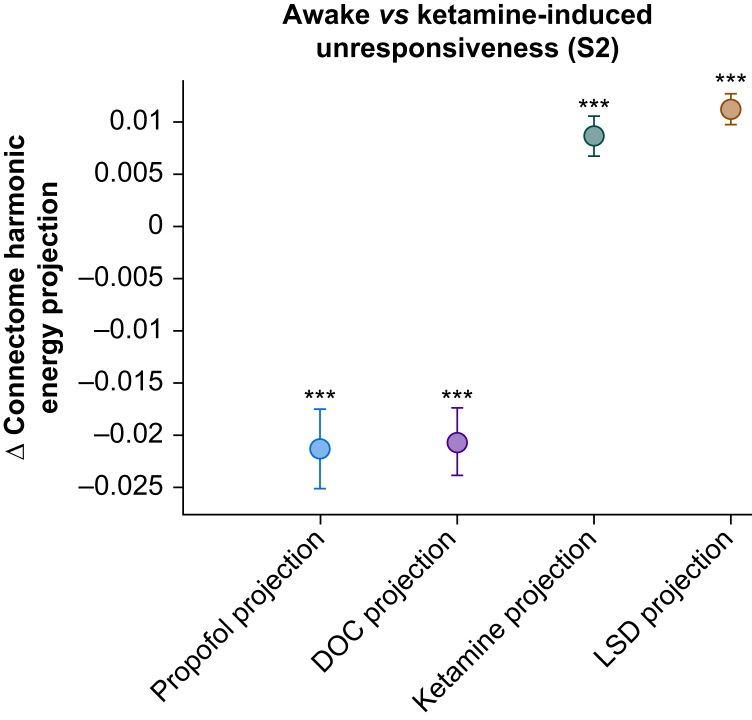
The connectome harmonic signature of ketamine-induced unresponsiveness aligns more with the signatures of psychedelic states. The figure displays the fixed effects and 95% confidence intervals (*N*=8) of different projections using the dot product between the multivariate connectome harmonic signature of ketamine-induced unresponsiveness (S2) *vs* wakefulness (Awake), and four other states identified by Luppi and colleagues^83^: propofol sedation *vs* wakefulness (blue), unresponsive *vs* responsive patients with DOC (violet), subanaesthetic ketamine *vs* placebo (orange), and LSD *vs* placebo (green). DOC, disorders of consciousness. ∗*p*<0.05, ∗∗*p*<0.01, *∗∗∗p*<0.001.

The multivariate connectome harmonic signature that best distinguished ketamine sedation (S2) from wakefulness coincides with the analogous signatures of LSD (Δ LSD–MVS projection =0.0113, *P*<0.001) and psychedelic ketamine (Δ ketamine–MVS projection =0.0087, *P*<0.001; Fig. 3). Conversely, the MVS that best discriminates between ketamine sedation (S2) and wakefulness is inversely related to the signatures of propofol sedation (Δ propofol–MVS projection =–0.0213, *P*<0.001; Fig. 3) and unresponsive *vs* responsive patients with DOC (Δ DOC–MVS projection =0.0206, *P*<0.001; Fig. 3). Similar results were found for the difference between ketamine light sedation (S1) and wakefulness (Awake) (Supplementary Fig. S5) and between the two different doses (S2 *vs* Awake) (Supplementary Fig. S6). This confirms earlier remarks that ketamine-induced unresponsiveness, such as atypical (subanaesthetic ketamine) and typical (LSD) psychedelics, gives rise to a more fine-grained organisation of brain activity, an observation that seems to increase with higher doses. In propofol sedation and in unresponsive patients with DOC, brain activity tends to be more coarse-grained,^83^ which corresponds to the misalignments between the MVSs presented here.

### Results are replicable with a different human structural connectome

Connectome harmonic signatures remain stable across scans of the same individuals in the same state of consciousness.^83^ This provides an important control for the results described previously. In addition, our results are not explained by motion artifacts of the participants (Supplementary Fig. S7) and do not depend on the specific operationalisation of the human connectome. After using an alternative method to reconstruct a high-resolution representation of the human connectome, combining a much larger sample of 985 HCP subjects, the results stay effectively the same (Supplementary Figs S8 and S9), making connectome harmonics a particularly suitable framework for mapping the landscape of consciousness across individuals and datasets.

### Multimodal integration: complexity/entropy obtained from combined functional MRI and EEG

The dataset we used contained simultaneous fMRI and EEG data, allowing us to relate outcomes of the CHD framework to more conventional EEG measures. This is important, because each imaging modality offers complementary insights into brain function (fMRI is an indirect measure of relative blood oxygenation, which is slower but provides superior spatial resolution; EEG measures aggregated electrical activity over the scalp with millisecond resolution). Their combination could provide a more comprehensive understanding of neural activity,^106^ especially in the context of consciousness and anaesthesia. It should be noted, however, that this last set of analyses is mainly exploratory.

We began by computing a measure of the temporal complexity (Lempel–Ziv compressibility, LZC) of the EEG signal. To see how global LZC changes during ketamine sedation, we averaged single-channel LZC over all available channels (Supplementary Fig. S1), and over all sliding calculation windows. Supplementary Fig. S10 shows the difference in whole-brain LZC values between conditions (Awake, S1, and S2). The results indicate a nonsignificant increase in EEG temporal complexity during ketamine sedation, which was similar to what we found with the repertoire entropy (RE) measure from CHD of the fMRI activity (Supplementary Fig. S4).

For the next step, we explored whether there were any topographical patterns in how LZC values related to ketamine sedation, rather than considering a single number aggregated across the entire scalp. No channels were significantly different between any of the conditions (Awake, S1, S2) after adjustment for multiple comparisons (Supplementary Fig. S11).^105^ However, between wakefulness (Awake) and both ketamine conditions (S1 and S2), a potential left-to-right gradient was observed, which was assessed by means of χ^2^ tests. For both (S2 minus Awake), χ^2^(1)=17.4107, *P*<0.001, and (S1 minus Awake), χ^2^(1)=17.7273, *P*<0.001, channel location (left, right) was significantly associated with the difference in LZC (diff ≥0, diff ≤0).

Subsequently, we correlated the whole-brain RE values (fMRI) with the global LZC values (EEG) across volunteers. Both measures assess a form of brain signal complexity, a concept associated with alterations in consciousness.^107^ We found no significant correlations between the two measures when comparing each pair of conditions (Supplementary Fig. S12).

**Fig 4:**
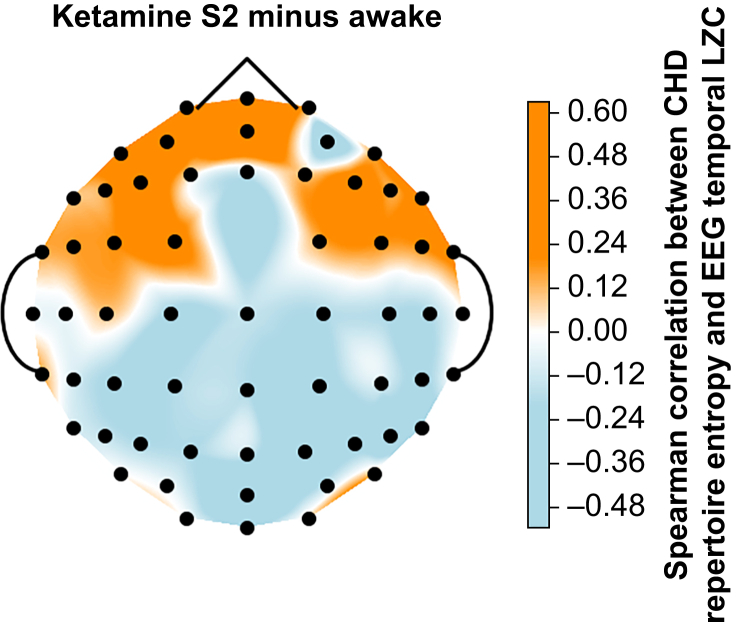
Channel-wise Spearman correlations between Δ whole-brain repertoire entropy (fMRI) and ΔLZC (EEG) (ketamine S2 minus awake). Channel-wise Spearman correlations between the difference in repertoire entropy (fMRI) and the difference in channel-specific LZC (EEG) for ketamine-induced unresponsiveness compared with wakefulness (*N=*8). The potential anterior-to-posterior gradient was assessed by χ^2^ test with electrode location (anterior, posterior) and correlation range (r <0, r ≥0) as variables, χ^2^(1)*=*29.8765, *P*<0.001. The middle row of electrodes was left out to add stringency to the test. CHD, connectome harmonic decomposition; fMRI, functional MRI; LZC, Lempel–Ziv complexity.

Lastly, we asked whether the change in EEG temporal complexity of any channel is related to the change in fMRI spatial complexity (CHD RE). We correlated the values of CHD RE (fMRI) with the channel-wise LZC values (EEG) across volunteers. As displayed in Fig. 4, we found no significant correlations between whole-brain RE values (fMRI) and channel-wise LZC values (EEG) when comparing wakefulness against ketamine-induced unresponsiveness (differences between the other conditions can be found in the Supplementary materials; Supplementary Fig. S13). However, we observed a possible anterior-to-posterior gradient in correlation values: positive at the front, but negative at the back of the brain (Fig. 4). We assessed the statistical significance of this observed pattern by means of a χ^2^ test. The central row of electrodes was left out to make the test more stringent, resulting in only two categories for ‘electrode location’ (anterior, posterior) and two categories for ‘correlation range’ (r <0, r ≥0). The association between the two variables was significant, χ^2^(1)*=*29.8765, *P*<0.001, which we interpreted as evidence for a significant gradient of correlations between local LZC and global CHD RE across individuals.

## Discussion

We investigated the spatial organisation of brain activity under ketamine-induced unresponsiveness using the recently developed framework of CHD (Fig. 1).^79^^,^^85^ Like the Fourier transform reflecting fundamental frequencies in temporal brain signals, the CHD method explicitly represents brain activity as fundamental harmonic modes by considering contributions from various spatial scales (granularities) of the underlying structural network (human connectome). Hence, CHD is in line with literature that considers brain-wide functional organisation to be informative of consciousness.^60^^,^^64^^,^^108^, ^109^, ^110^, ^111^, ^112^, ^113^, ^114^, ^115^, ^116^, ^117^, ^118^, ^119^

We have shown that ketamine-induced unresponsiveness increases the contribution of fine-grained (more granular) connectome harmonics and decreases the contribution of coarse-grained (less granular) harmonics, a pattern reminiscent of altered states of consciousness induced by psychedelics, subanaesthetic doses of ketamine, and meditation.^80^, ^81^, ^82^, ^83^^,^^85^ At first glance, this might seem odd, because the volunteers in this dataset appeared unconscious and were completely unresponsive to instructions or questions.^88^ One factor that could explain the resemblance of the CHD signature to that of altered states of consciousness is the occurrence of ‘psychedelic-like’ dreams in all participants during the unresponsive period (Table S1), a finding that aligns with previous structured reports from a study investigating ketamine sedation.^25^ These observations are not unique to the specific sample or administration method used in this dataset, as they have been documented in different contexts.^25^^,^^43^, ^44^, ^45^, ^46^

A substantial body of evidence has observed another shared characteristic between classical serotonergic psychedelics and ketamine, namely an increase in long-range/internetwork FC.^120^, ^121^, ^122^, ^123^ These observations are consistent with the results obtained by CHD. Firstly, CHD pertains to functional *activity* rather than functional *connectivity* (FC), which reflects the correlation between regional time series.^124^ FC provides information about which brain regions co-fluctuate over time, whereas CHD provides an instantaneous measure of how granular brain activity is across the cortex, using basic patterns (connectome harmonics) obtained from the underlying structural network organisation (human connectome), but without focusing on any specific brain region. These connectome harmonics are organised from less granular (coarse-grained) to more granular (fine-grained). The granularity of the functional activity pattern at any given moment, obtained from fMRI, thus depends on which connectome harmonics contribute most to the signal.

Although the results from CHD and FC analyses reflect different aspects of brain functioning, they are consistent. In the brain, structural connections broadly follow an exponential distance rule (EDR), such that regions that are further away tend to have weaker anatomical connections (although important long-range exceptions can also be found).^125^^,^^126^ In broad terms (though not without exceptions), FC correlates with SC^127^^,^^128^ such that stronger FC occurs for stronger SC. So, an increase in FC between far-away regions, as seen under the influence of classical psychedelics and ketamine, is consistent with decoupling of FC from SC as a result of the known relationship between SC and distance (which would predict relatively weaker FC for regions that are far away). A functional decoupling from SC is also what we observed, given the increased prevalence of fine-grained harmonics, which have been interpreted as reflecting a structurally decoupled signal.^83^^,^^96^

Ketamine's similarity to classical psychedelics is further underscored by their shared ability to alter neuroplasticity,^129^^,^^130^ induce similar phenomenology,^131^ increase or maintain neurophysiologic complexity,^23^^,^^132^ and expand the repertoire of functional brain activity models.^24^^,^^83^ Other clear evidence of similarity between ketamine-induced unresponsiveness and states induced by classical psychedelics was given by Luppi and colleagues.^133^ This study used *in vivo* maps of neurotransmitter distribution in the human brain, obtained from positron emission tomography, to examine the macroscale neurotransmitter signatures of a wide range of pharmacological agents. They found that both ketamine and serotonergic psychedelics (e.g. LSD, psilocybin) showed similar patterns of functional reorganisation in the brain, indicating that their neurotransmitter profiles align with molecular and subjective effects, rather than with behavioural effects, a finding we confirmed.

The second main finding is that CHD can, as previously suggested,^83^ be used to track consciousness in the absence of behavioural awareness. To date, the perturbational complexity index (PCI) is considered one of the most sensitive indicators to the presence or absence of consciousness *per se*,^33^^,^^107^ showing almost perfect discrimination between unconsciousness (non-rapid eye movement [NREM] sleep, anaesthesia with midazolam, propofol, and xenon) and consciousness (wakefulness), even if disconnected (ketamine sedation, dreaming, psilocybin-induced psychedelic states) or in patients with DOC.^25^^,^^33^^,^^134^^,^^135^ PCI is derived by applying transcranial magnetic stimulation to a small region of the cerebral cortex and recording the resulting spatiotemporal activity across broader cortical areas using high-density EEG. Therefore, due to its interventional nature, PCI cannot be applied retrospectively to existing data. CHD, on the other hand, can overcome this limitation, as the framework is predicated only on fMRI data and is thus suitable for retrospective analysis. Additionally, whereas PCI considers temporal complexity of the signal's spatial propagation following perturbation, resulting in a spatiotemporal complexity value, CHD provides a spatial complexity measure on a much finer scale because of the properties of fMRI. Another key difference is that CHD provides information about the relationship between brain structure and function, and how structure (network architecture) shapes activity across different scales. PCI does not address structure–function relationships. Lastly, PCI provides a one-dimensional output, whereas CHD provides multidimensional information about the spatial organisation (granularity) of brain activity. For these reasons, the two measures are distinct both in what modality they depend on, what they measure, and what insights they provide about the brain, and we believe that they should be viewed as complementary rather than as alternatives. We therefore welcome future studies that explicitly investigate their relationship within the same individuals.

Thirdly, our findings also confirm the crucial role played by fine-grained harmonics in distinguishing between different states of consciousness.^80^^,^^83^ This valuable insight was made possible by leveraging high-quality, high-resolution diffusion data from the HCP, allowing to achieve resolutions up to three orders of magnitude finer than other methods of harmonic mode decomposition relying on parcellated data.^41^^,^^70^^,^^75^^,^^96^^,^^136^^,^^137^ The results were successfully replicated using a high-resolution connectome constructed from data collected from 985 HCP subjects, representing one of the most comprehensive characterisations of the SC of the human brain to date.^83^

Obtaining high-quality connectome reconstructions can present challenges. Firstly, variability in data quality, arising from factors such as imaging artifacts, motion during scanning, and signal-to-noise ratio, can introduce inaccuracies in the reconstructed connectome.^138^ Secondly, the human brain exhibits significant intersubject variability in anatomy and connectivity patterns. Accounting for this variability and establishing reliable norms for connectome reconstruction across individuals is challenging.^139^ Thirdly, combining data from multiple imaging modalities, such as structural MRI, diffusion MRI, and fMRI, poses challenges in terms of data fusion and integration. Aligning data from different modalities to construct a comprehensive connectome requires sophisticated processing and validation methods.^140^ Fourthly, diffusion imaging and tractography cannot accurately infer fibre directionality, a crucial aspect of brain wiring.^141^ Lastly, all molecular and cellular components of the human structural connectome undergo constant remodelling, replacement, and resynthesis,^142^ a phenomenon known as *structural plasticity*.^143^ Each reconstructed connectome thus captures only a snapshot of a dynamic process, potentially complicating the reliable inference of structure–function relationships. Taking into account these limitations, the connectome used here stands out as one of the most thorough descriptions of the structure within the human brain to date, adding credibility to our findings.

Pang and colleagues^144^ argued that ‘structural eigenmodes (harmonic modes) derived solely from the brain's geometry provide a more compact, accurate and parsimonious representation of its macroscale activity than alternative connectome-based models'. However, Faskowitz and colleagues^145^ countered that this claim contradicts prevailing theories regarding information flow in the brain, highlighting the significance of long-range axonal connections and bundled white matter in facilitating signal relay among cortical areas.^72^^,^^146^^,^^147^ Despite these controversies, a recent study was able to demonstrate that, connected through the EDR, cortical short-range connectivity and geometry are related in being spatially embedded, structural connections implicitly encoding geometry, with an additional important role of long-range connectivity exceptions to the EDR.^126^ Thus, the best results in explaining functional activity patterns in terms of underlying eigenmodes are obtained by incorporating both short-range connectivity via the EDR and long-range connectivity embodied by the brain's white matter tracts, adding merit to the framework used in the current study.

Another limitation is the rather low number of participants included in this study. With a small sample size, statistical power decreases, making it more difficult to detect real effects or relationships between variables.^148^ Nevertheless, the fact that our results are statistically robust do not correlate with motion parameters (Supplementary Fig. S7), were generalised across different connectomes (HCP10 and HCP985; Supplementary Figs S8 and S9), and align with previous investigations mitigate some of these shortcomings. The number of volunteers used in the current investigation falls within the range of most published fMRI studies examining the effects of pharmacological perturbations on brain activity (between 7 and 19).^22^, ^23^, ^24^, ^25^, ^26^^,^^28^^,^^31^^,^^32^^,^^34^^,^^88^^,^^108^^,^^109^ Nonetheless, we recognise the importance of replicating these findings in future studies using a similar ketamine dose but with a larger sample. It would also be interesting to look at the CHD signatures of complete *unconsciousness* induced by ketamine, as not everyone reports the episodes of disconnected consciousness,^46^^,^^149^ and compare them with the CHD signatures presented here. To our knowledge, the only two fMRI datasets available that also include subjective reports found episodes of disconnected consciousness in all participants undergoing ketamine sedation.^25^^,^^88^

Finally, the current dataset provided simultaneous fMRI and EEG data, which allowed us to compare the novel complexity measure pertaining to CHD, RE (Supplementary Fig. S4) with the more widely used complexity measure obtained from EEG data, LZC. Firstly, we replicated previous research suggesting that ketamine-induced unresponsiveness maintains or even increases global spatiotemporal EEG complexity in humans (Supplementary Fig. S10).^23^^,^^25^^,^^41^ However, there were positive values (increased LZC) on the left side of the brain, and negative values (decreased LZC) on the right side during ketamine sedation compared with wakefulness (S1/S2 minus Awake). We are not aware of similar LZC gradients, but as this dataset consisted only of right-handed volunteers, we speculate that the effects of ketamine on LZC might be lateralised.

Within the same individuals, we found no significant linear association between the complexity measure pertaining to fMRI, RE obtained from CHD, and the complexity measure pertaining to EEG, global LZC, when comparing all three conditions (Awake, S1, and S2) (Supplementary Fig. S12). Reasons for this might be that they measure different forms of complexity, spatial for RE and spatiotemporal for LZC, or come from the distinct imaging modalities, fMRI and EEG. A recent preprint investigating the classical psychedelic DMT did find a positive correlation between the two measures, showing multimodal convergence.^85^ With more subjects, we hope to see this convergence with ketamine as well. Analysis of individual electrode LZC values, on the contrary, revealed a positive linear correlation with RE in anterior electrodes and a negative correlation in posterior electrodes, resulting in a significant anterior-to-posterior gradient. The same gradient was not observed when comparing other pairs of conditions, confirming previous conclusions that ketamine induces very complex dose-dependent EEG dynamics that are hard to systematise.^150^

Given that our findings confirm the previous conclusion that CHD seems to reliably track consciousness in the absence of behavioural responsiveness, it could serve as a valuable tool in clinical settings to identify patients with DOC who warrant further assessment. This screening tool could help reduce the rate of misdiagnosis observed in these patients with DOC when relying solely on behavioural criteria.^151^^,^^152^ In addition, CHD might offer valuable insights in addressing challenges during general anaesthesia, such as finding the optimal level of (un)consciousness.^153^ Replicating these findings in diverse and larger samples of both patients with DOC and individuals undergoing pharmacological perturbations of consciousness is essential to validate their reliability and robustness. Lastly, to make our findings clinically relevant, a more individualised approach will need to be taken, as the substantial interindividual variability in response to ketamine has been presented.^154^

In conclusion, the robust analytical approach of CHD was used to investigate the brain dynamics of ketamine-induced unresponsiveness, unveiling fresh insights into the neural underpinnings of this unique state and its relationship to human consciousness. The findings underscore the importance of considering global brain function and associated subjective experiences in terms of the dynamic activation of harmonic brain states (connectome harmonics). Notably, this perspective reveals the dynamic spectrum of brain activity and suggests a shift to more fine-grained functional organisation of brain activity during ketamine-induced unresponsiveness compared with wakefulness, possibly indicative of decoupling between structure and function. This particular signature maps well onto the signatures seen for psychedelic drugs (DMT, LSD, and psilocybin) and subanaesthetic ketamine, which can be related to the psychedelic-like dream reports of the volunteers during the sedative period. Overall, our results align with the previous hypothesis that CHD analysis can be used to track consciousness in participants who appear unconscious when assessed based on behavioural responsiveness.

## Authors’ contributions

Conception: MVM, EAS, AIL

Supervision: EAS, AIL

Data extraction: VB, AV

Data analysis and interpretation: MVM, JV, SA, PC, NA, AIL

Drafting the original manuscript: MVM

Revising subsequent drafts: all authors

Approval of the final manuscript: all authors

## Funding

10.13039/100007631Canadian Institute for Advanced Research (grant RCZB/072 RG93193 to EAS); Stephen Erskine Fellowship at Queens' College, Cambridge (to EAS); Belgian National Funds for Scientific Research (to PC and NLNA); GIGA-Doctoral School for Health Sciences (University of Liège) (to PC); Human Brain Project (to NLNA); EU H2020 Future and Emerging Technologies (FET) Proactive project Neurotwin (grant agreement no. 101017716 to JV); European Research Council Consolidator Grant: CAREGIVING (615539), Pettit Foundation, Carlsberg Foundation and Center for Music in the Brain, funded by the Danish National Research Foundation (DNRF117 to MLK); Engineering and Physical Sciences Research Council (capital grant EP/T022159/1); DiRAC funding from the Science and Technology Facilities Council; MRC research infrastructure award (MR/M009041/1).

## Data availability

The raw ketamine data are available upon request from author VB (Vincent.Bonhomme@uliege.be).

The code for the connectome harmonic and EEG analyses is available upon request from authors MVM and AIL (mv554@cam.ac.uk and al857@cam.ac.uk, respectively).

## Declaration of interests

VB has had financial relationships with the following companies: Orion Pharma, Medtronic, Edwards, and Elsevier. All other authors declare they have no competing interests.
