# Supplementary material for: Connectome harmonic decomposition tracks the presence of disconnected consciousness during ketamine-induced unresponsiveness
Source: Br J Anaesth. 2025 Feb 10;134(4):1088–104. doi: 10.1016/j.bja.2024.12.036 (PMC11947573; doi:10.1016/j.bja.2024.12.036)
Supplement: Multimedia component 1 [file mmc1.docx]

**
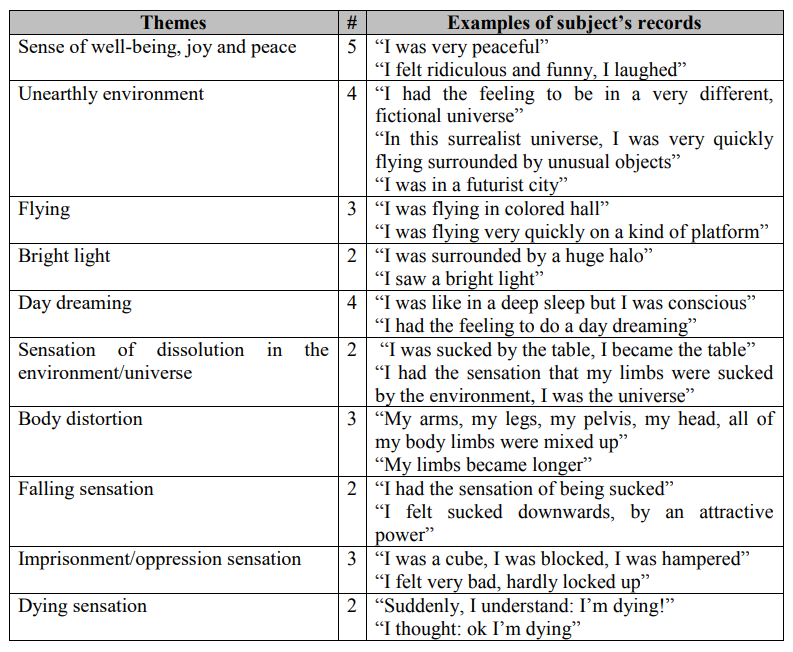
Supplementary Materials**

**Table S1. Themes of the dreams volunteers had during ketamine sedation.**

Major cognitive themes perceived and recalled by volunteers during ketamine infusion and whose data were included in the analysis.

The column headed by # gives the number among eight subjects having experienced the concerned theme.

This table was adapted from Bonhomme and colleagues,^1^ and used with permission.


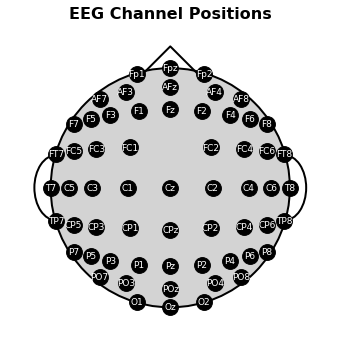


**Figure S1. Electroencephalogram (EEG) channel locations with their corresponding names.**


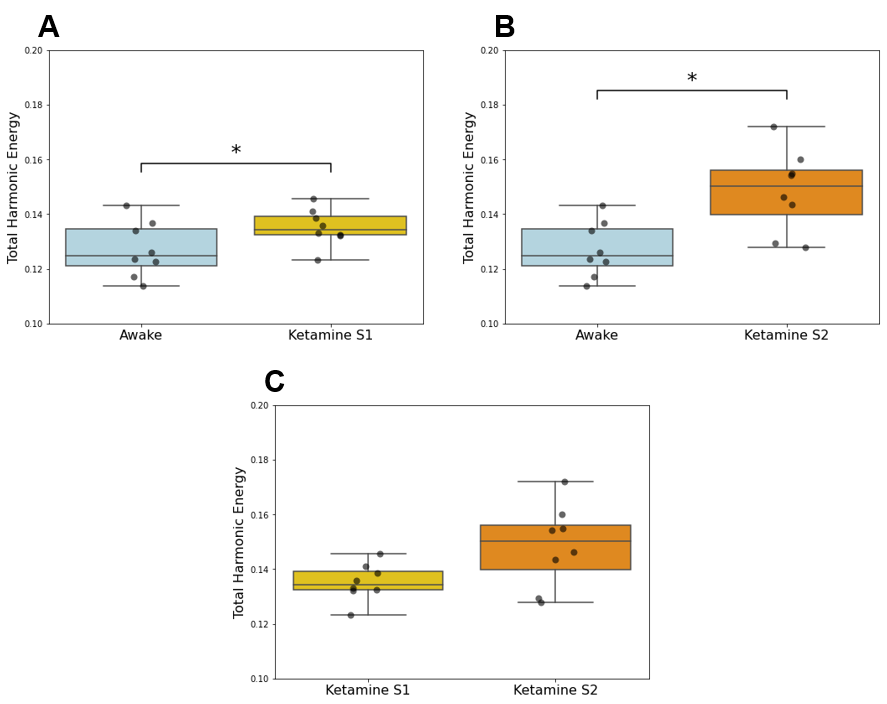


**Figure S2. Total harmonic energy of brain activity under ketamine sedation.**

**A.** The difference in total energy across all connectome harmonics (n = 18,715) between wakefulness [Awake] and ketamine light sedation [S1], *t*(14) = 3.2204, *p* = 0.0146 (*N* = 8, repeated-measures t-test). **B.** The difference in total energy of all connectome harmonics between wakefulness [Awake] and ketamine-induced unresponsiveness [S2], *t*(14) = 2.8208, *p* = 0.0257 (*N* = 8, repeated-measures t-test). **C.** The difference in total energy of all connectome harmonics between ketamine light sedation [S1] and ketamine-induced unresponsiveness [S2], *t*(14) = 2.0563, *p* = 0.0788 (*N* = 8, repeated-measures t-test).

* *p* < 0.05


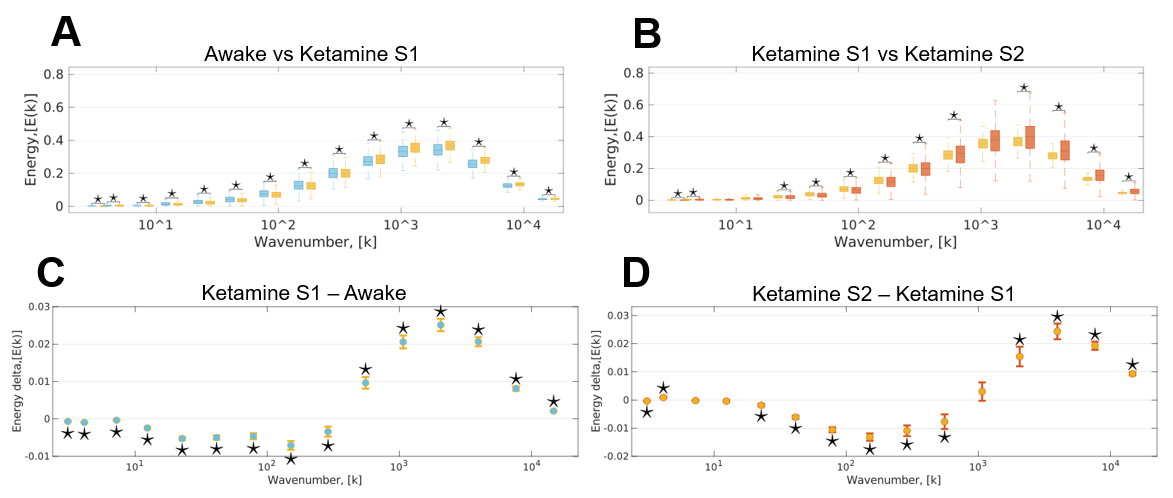


**Figure S3. Connectome harmonic energy signature of ketamine sedation.**

**A.** The binned energy spectrum across subjects and timepoints (*N* = 8, 300 timepoints each), with ketamine light sedation [S1] as target state and wakefulness [Awake] as reference state. **B.** The binned energy spectrum across subjects and timepoints (*N* = 8, 300 timepoints each), with ketamine-induced unresponsiveness [S2] as target state and ketamine light sedation [S1] as reference state. **C.** Statistical estimates from LME modelling between wakefulness [Awake] and ketamine light sedation [S1], treating condition as a fixed effect and subjects as random effects. Timepoints were also incorporated as random effects, nested within subjects. **D.** Statistical estimates from LME modelling between ketamine light sedation [S1] and ketamine-induced unresponsiveness [S2], treating condition as a fixed effect and subjects as random effects. Timepoints were also incorporated as random effects, nested within subjects.

Characteristics of the boxplots are as follows: central line indicates the median, box edges represent 25^th^ and 75^th^ percentiles, whiskers resemble 1.5x inter-quartile interval. This is consistent with the data presented before by Luppi and colleagues.^2^

**p* < 0.05, FDR-corrected across 15 frequency bins.


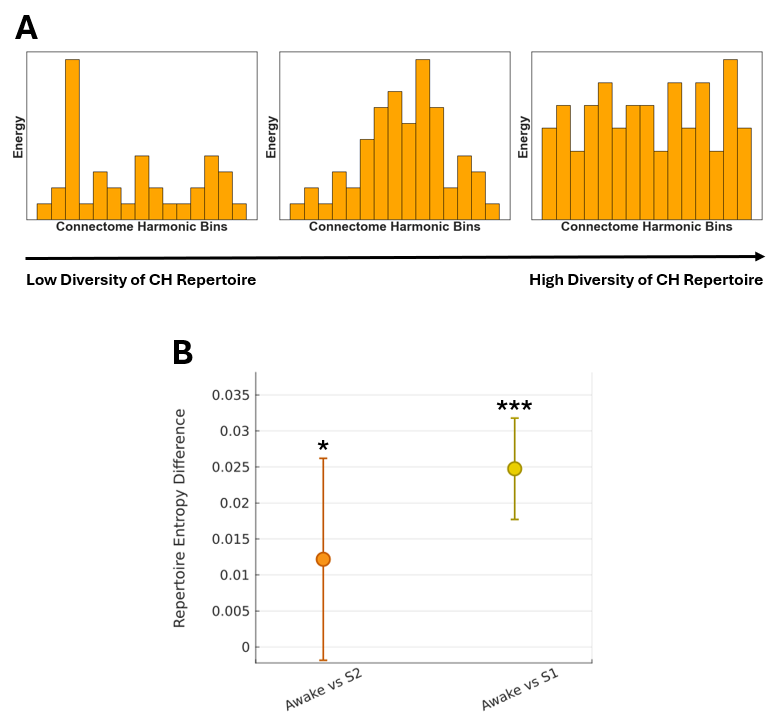


**Figure S4. Diversity of connectome harmonic repertoire of ketamine sedation.**

**A.** Possible connectome harmonic repertoires from low (dominated by a specific range of spatial frequencies) to high (approaching a uniform distribution of spatial frequencies) diversity. See Methods for calculation. **B.** Pairs of conditions were compared with linear mixed effects modelling, treating condition as a fixed effect and subjects as random effects (*N* = 8, 300 timepoints). The figure displays the fixed effects (and 95% confidence intervals) between conditions. Ketamine-induced unresponsiveness [S2] minus wakefulness [Awake], *p* = 0.0889. Ketamine light sedation [S1] minus wakefulness [Awake], *p* < 0.001.

****p* < 0.001, ***p* < 0.01, **p* < 0.1


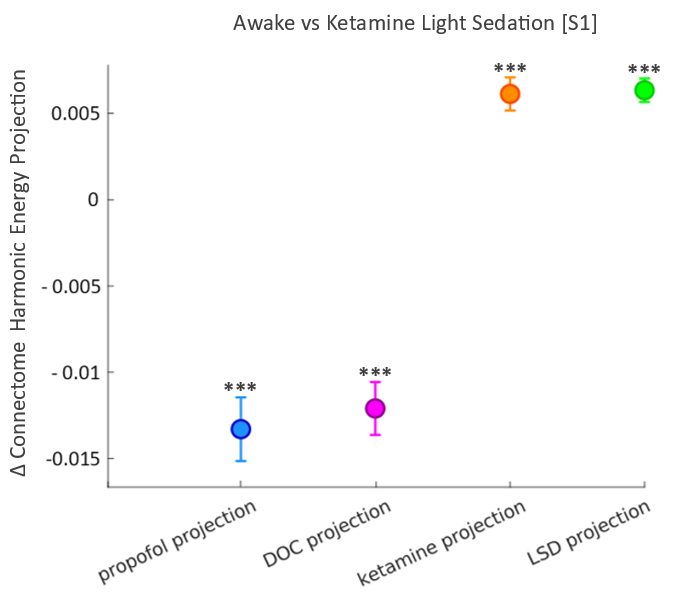


**Figure S5. Alignments of the connectome harmonic signature of ketamine light sedation [S1] compared to wakefulness [Awake] with other altered brain states.**

The figure displays the fixed effects and 95% confidence intervals (*N* = 8) of different projections – using the dot-product – between the multivariate connectome harmonic signature of ketamine light sedation [S1] versus wakefulness [Awake], and four other states previously identified by Luppi and colleagues^2^: propofol sedation versus wakefulness (blue), unresponsive versus responsive DOC patients (violet), ketamine sub-anaesthesia versus placebo (orange), and LSD versus placebo (green).

**p* < 0.05, ***p* < 0.01, ****p* < 0.001


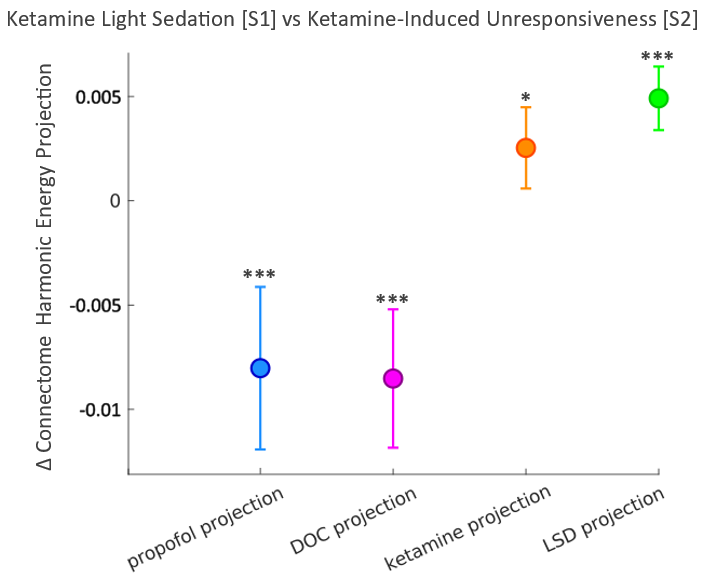


**Figure S6. Alignments of the connectome harmonic Signature of ketamine-induced unresponsiveness [S2] compared to ketamine light sedation [S1] with other altered brain states**

The figure displays the fixed effects and 95% confidence intervals (*N* = 8) of different projections – using the dot-product – between the multivariate connectome harmonic signature of ketamine light sedation [S1] versus ketamine-induced unresponsiveness [S2], and four other states previously identified by Luppi and colleagues^2^: propofol sedation versus wakefulness (blue), unresponsive versus responsive DoC patients (violet), ketamine sub-anaesthesia versus placebo (orange), and LSD versus placebo (green).

**p* < 0.05, ***p* < 0.01, ****p* < 0.001


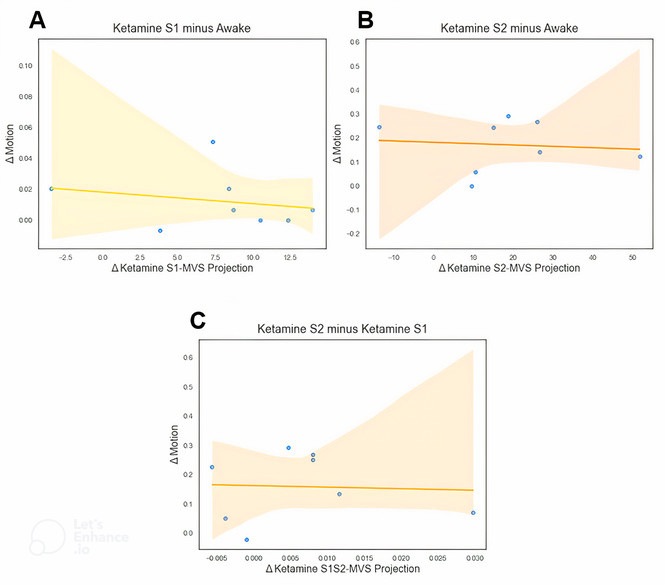


**Figure S7. Correlations between the projections of individual connectome harmonic signatures onto multivariate energy signature (MVS) and head motion of the volunteers.**

**A.** Delta in ketamine light sedation [S1] connectome harmonic energy projection onto the MVS discriminating between wakefulness [Awake] and ketamine light sedation [S1], versus the delta in head motion [S1 minus Awake], *r_s_*(6) = -0.27, *p* = 0.53 (*N* = 8). **B.** Delta in ketamine-induced unresponsiveness [S2] connectome harmonic energy projection onto the MVS discriminating between wakefulness [Awake] and ketamine-induced unresponsiveness [S2], versus the delta in head motion [S2 minus Awake], *r_s_*(6) = 0.14, *p* = 0.74 (*N* = 8). **C.** Delta in ketamine-induced unresponsiveness [S2] connectome harmonic energy projection onto the MVS discriminating between ketamine light sedation [S1] and ketamine-induced unresponsiveness [S2], versus the delta in head motion [S2 minus S1], *r_s_*(6) = 0.14, *p* = 0.74 (*N* = 8) .


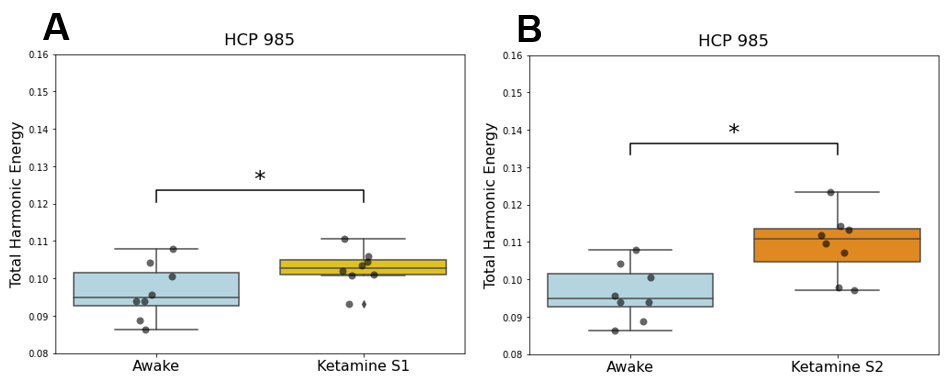


**Figure S8. Total harmonic energy of brain activity under ketamine sedation, for the HCP-985 connectome.**

**A.** The difference in total energy of all connectome harmonics (n = 18,715) between wakefulness [Awake] and ketamine light sedation [S1], *t*(14) = 3.6838, *p* = 0.0078 (*N* = 8, repeated-measures t-test). **B.** The difference in total energy of all connectome harmonics between wakefulness [Awake] and ketamine-induced unresponsiveness [S2], *t*(14) = 2.7008, *p* = 0.0306 (*N* = 8, repeated-measures t-test).

* *p* < 0.05


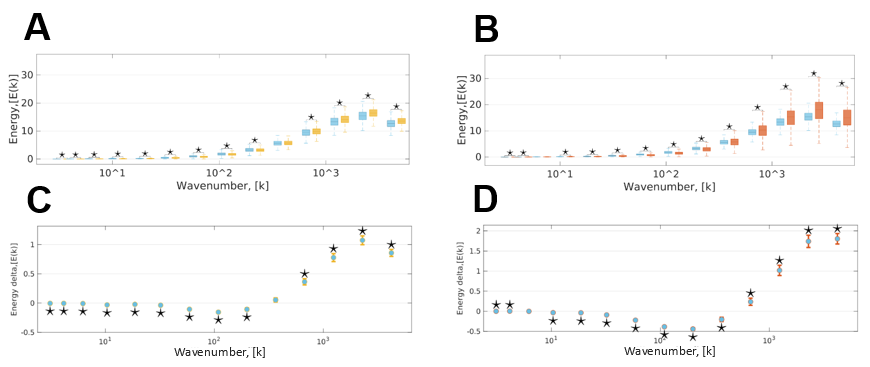


**Figure S9. Connectome harmonic energy signature of ketamine light sedation [S1] and ketamine-induced unresponsiveness [S2], for the HCP-985 connectome.**

**A.** The binned energy spectrum across subjects and timepoints (*N* = 8, 300 timepoints each), with ketamine light sedation [S1] as target state and wakefulness [Awake] as reference state. **B.** The binned energy spectrum across subjects and timepoints (*N* = 8, 300 timepoints each), with ketamine-induced unresponsiveness [S2] as target state and wakefulness [Awake] as reference state. **C.** Statistical estimates from LME modelling between wakefulness [Awake] and ketamine light sedation [S1], treating condition as a fixed effect and subjects as random effects. Timepoints were also incorporated as random effects, nested within subjects. **D.** Statistical estimates from LME modelling between wakefulness [Awake] and ketamine-induced unresponsiveness [S2], treating condition as a fixed effect and subjects as random effects. Timepoints were also incorporated as random effects, nested within subjects.

Characteristics of the boxplots are as follows: central line indicates the median, box edges represent 25^th^ and 75^th^ percentiles, whiskers resemble 1.5x inter-quartile interval. This is consistent with the data presented before by Luppi and colleagues.^2^

**p* < 0.05, FDR-corrected across 15 frequency bins.

**
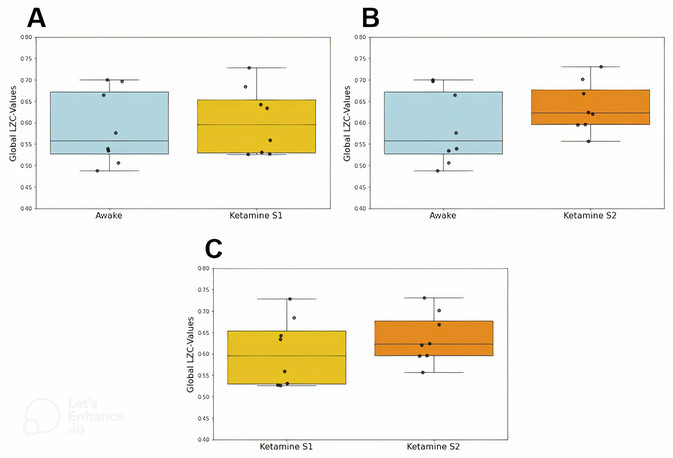
**

**Figure S10. Global Lempel-Ziv Complexity (LZC) values, averaged over electrodes.**

**A.** The difference in LZC, averaged over electrodes (n = 60), between wakefulness [Awake] and ketamine light sedation [S1], *t*(14) = 0.5454, *p* = 0.6024 (*N* = 8, repeated-measures t-test). **B.** The difference in LZC, averaged over electrodes (n = 60), between wakefulness [Awake] and ketamine-induced unresponsiveness [S2], *t*(14) = 1.7664, *p* = 0.1207 (*N* = 8, repeated-measures t-test). **C.** The difference in LZC, averaged over electrodes (n = 60), between ketamine light sedation [S1] and ketamine-induced unresponsiveness [S2], *t*(14) = 1.6223, *p* = 0.1488 (*N* = 8, repeated-measures t-test).

* *p* < 0.05

**Figure S11. Channel-wise differences in LZC between conditions.**

**A.** Channel-wise differences in LZC between wakefulness [Awake] and ketamine light sedation [S1] (*N* = 8, repeated-measures t-tests). The potential left-to-right gradient was assessed by means of a chi-squared test with electrode location (left, right) and LZC difference (diff ≥ 0, diff ≤ 0) as variables, *Χ²*(1) *=* 17.7273, *p* < 0.001. The middle row of electrodes was left out to add stringency to the test. **B.** Channel-wise differences in LZC between wakefulness [Awake] and ketamine-induced unresponsiveness [S2] (*N* = 8, repeated-measures t-tests). The potential left-to-right gradient was assessed by means of a chi-squared test with electrode location (left, right) and LZC difference (diff ≥ 0, diff ≤ 0) as variables, *Χ²*(1) *=* 17.4107, *p* < 0.001. The middle row of electrodes was left out to add stringency to the test. **C.** Channel-wise differences in LZC between ketamine light sedation [S1] and ketamine-induced unresponsiveness [S2] (*N* = 8, repeated-measures t-tests). No potential gradient was observed, so no additional analyses were conducted.

White circles mark electrodes in which the LZC difference was significant (*p* < 0.05, uncorrected). No significant electrodes survived correction for multiple comparisons by means of the Benjamini-Hochberg method.^3^


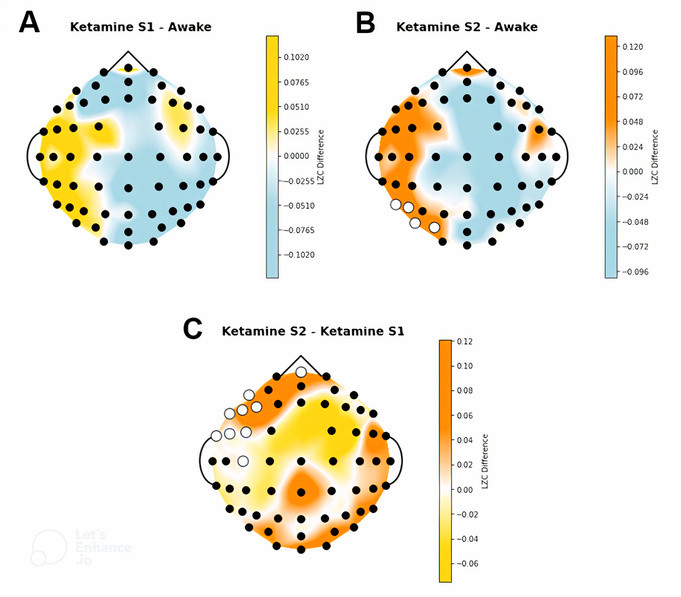

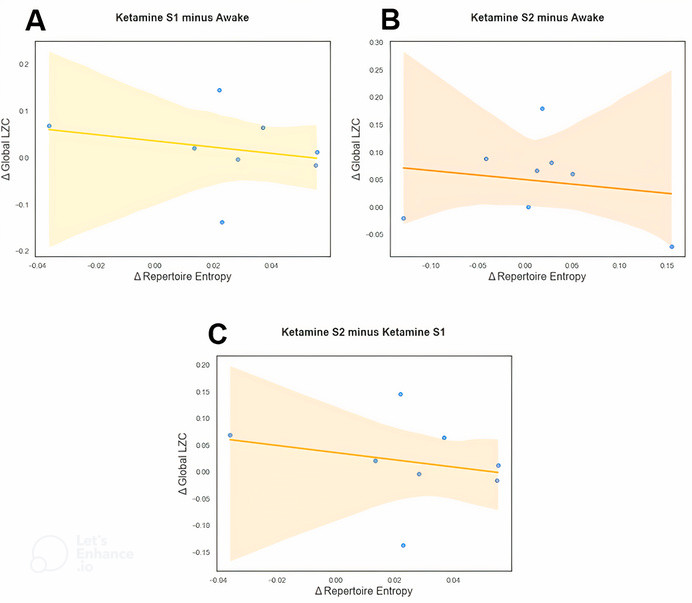


**Figure S12. Spearman correlations between Δ Repertoire Entropy and Δ Global LZC.**

**A.** Spearman correlation between Δ Repertoire Entropy and Δ Global LZC [S1 minus Awake], *r_s_*(6) = -0.4761, *p* = 0.2329 (*N* = 8). **B.** Spearman correlation between Δ Repertoire Entropy and Δ Global LZC [S2 minus Awake], *r_s_*(6) = -0.1190, *p* = 0.7789 (*N* = 8). **C.** Spearman correlation between Δ Repertoire Entropy and Δ Global LZC [S2 minus S1], *r_s_*(6) = 0.2143, *p* = 0.6103 (*N* = 8).

**
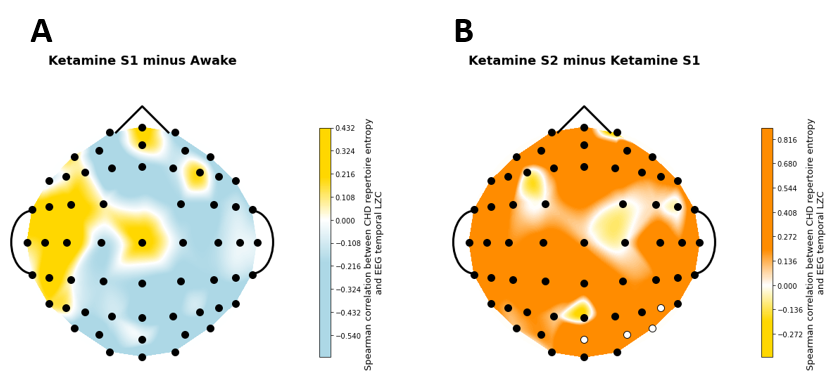
**

**Figure S13. Channel-Wise Spearman Correlations Between Δ Whole-Brain Repertoire Entropy (fMRI) and ΔLZC (EEG).**

**A.** Channel-wise spearman correlations (*N* = 8) between the difference in repertoire entropy (fMRI) and the difference in channel-specific LZC (EEG) [S1 minus Awake]. The left-to-right gradient was assessed by a chi-squared test with electrode location (left, right) and correlation range (r < 0, r ≥ 0) as variables, *Χ²*(1) *=* 15.2569, *p* < 0.001. The middle column of electrodes was left out to add stringency to the test. **B.** Channel-wise spearman correlations (*N* = 8) between the difference in repertoire entropy (fMRI) and the difference in channel-specific LZC (EEG) [S2 minus S1]. No potential gradient was observed in this figure, so no further chi-squared test was performed.

White circles mark electrodes in which the spearman correlation was significantly different from zero (*p* < 0.05, uncorrected). No significant electrodes survived correction for multiple comparisons by means of the Benjamini-Hochberg method.^3^

**References**

1. Bonhomme V, Vanhaudenhuyse A, Demertzi A, et al. Resting-state network-specific breakdown of functional connectivity during ketamine alteration of consciousness in volunteers. Anesthesiology 2016; 125(5): 873–88
2. Luppi AI, Vohryzek J, Kringelbach ML, et al. Distributed harmonic patterns of structure-function dependence orchestrate human consciousness. Commun Biol 2023; 6: 117
3. Benjamini Y, Hochberg Y. Controlling the false discovery rate: A practical and powerful approach to multiple testing. J R Stat Soc 1995; 57(1): 289-300
